# Effects of pulmonary rehabilitation on diaphragm thickness and contractility in patients with chronic obstructive pulmonary disease

**DOI:** 10.3906/sag-2105-345

**Published:** 2021-09-27

**Authors:** Seçilay GÜNEŞ, Aysun GENÇ, Yesim KURTAİŞ AYTÜR, Fatma ÇİFTÇİ, Serhat HAYME, Akın KAYA

**Affiliations:** 1 Department of Physical Medicine and Rehabilitation, Faculty of Medicine, Ankara University, Ankara, Turkey; 2 Department of Chest Diseases, Faculty of Medicine, Ankara University, Ankara, Turkey; 3 Department of Biostatistics, Faculty of Medicine, Ankara University, Ankara, Turkey

**Keywords:** Chronic obstructive pulmonary disease, pulmonary rehabilitation, diaphragm thickness, diaphragm contractility, ultrasonography

## Abstract

**Background/aim:**

Studies are showing that pulmonary rehabilitation (PR) increases diaphragmatic excursion by decreasing hyperinflation in patients with chronic obstructive pulmonary disease (COPD). However, there is a lack of knowledge about its effects on the diaphragm thickness (dt) and contractility. This study aims to evaluate the dt and contractility before and after PR in patients with COPD.

**Materials and methods:**

All subjects participated in an out-patient PR of 6 weeks and 3 sessions per week prospectively. Dyspnea severity, the disease-specific quality of life (St. Georges Respiratory Questionnaire-SGRQ), pulmonary function tests (PFT), exercise capacity, the dt at the end of the expiration and at maximal inspiration (B-mode ultrasound) were evaluated at baseline and after PR.

**Results:**

A total of 34 patients with a mean age and FEV1 61.05 ± 8.22 years and 57.9 ± 20.4% predicted respectively showed improvements in exercise capacity and some items of PFT and SGRQ. Diaphragmatic thickness at the end of the expiration also significantly improved regardless of the disease severity and was positively correlated with functional performance. The 6-weeks of PR didn’t result in a significant difference in diaphragm contractility.

**Conclusion:**

The 6-weeks of PR resulted in a significant increase in dt without a significant difference in diaphragm contractility.

## 1. Introduction

Chronic obstructive pulmonary disease (COPD) is characterized by progressive airflow restriction due to chronic inflammatory response in the airways [1]. The airflow restriction results in dynamic lung hyperinflation, which leads to diaphragm shortening and causes the mechanical disadvantage of the diaphragm [2]. Oxidative stress and the systemic inflammatory process leading to general muscular atrophy and diaphragmatic alterations [3], such as contractile protein wasting or fiber-type shift, contribute to diaphragmatic dysfunction [4].

The assessment of the diaphragmatic function in COPD patients has been the focus of many studies. Trans-diaphragmatic pressure is the main parameter in the assessment of diaphragmatic dysfunction [5]. However, this method is invasive and uncomfortable for the patient. Thus, ultrasonographic evaluation of the diaphragm, which is non-invasive, has become popular recently. While M-mode ultrasound allows the evaluation of the diaphragmatic motion, B-mode ultrasound provides significant information about the structure and contractility of the diaphragm by measuring the thickness of the muscle and the thickening ratio during respiration [6]. It was reported that the ultrasonographic diaphragm thickness was significantly correlated with the trans-diaphragmatic pressure [7]. To date, many studies were conducted to evaluate the diaphragm thickness in patients with COPD using ultrasonography [5,8,9]. In one study, reduced diaphragm thickness compared to healthy adults was shown [5], which was considered as an indicator of diaphragm dysfunction; however, no difference was observed in another study [9].

Pulmonary rehabilitation (PR), a multicomponent intervention based on individually tailored exercise training, education, dietary advice, psychological and behavioral intervention, has emerged as arguably the most effective non-pharmacological treatment in improving exercise capacity, dyspnea, and quality of life in patients with COPD [10,11]. PR has also been shown to improve skeletal muscle dysfunction in these patients; however, its effects on the diaphragm function, which is also a striated muscle, are not well known and have been a subject of research in recent years [12,13]. In studies evaluating the effects of PR on diaphragm function by sonography, it was shown that diaphragmatic mobility could improve after PR [12]. However, there is no study evaluating the effects of PR on diaphragm thickness and contractility in patients with COPD.

We hypothesized that the exercise program of PR may increase the thickness and contractility of the diaphragm. To test this hypothesis, the primary aim of this study was to evaluate the diaphragm thickness and contractility before and after PR in patients with COPD. The secondary aim was to discern whether there was a relationship between diaphragm thickness changes and dynamic PFT outcomes and exercise capacity of patients after PR.

## 2. Materials and methods

### 2.1. Subjects

Adult patients who were diagnosed with COPD at Ankara University Faculty of Medicine, Department of Chest Diseases and referred to the Department of Physical Medicine and Rehabilitation for PR were included. Patients who were between the age of 18–80 years did not previously receive PR in the last 6 months were included. Patients with known neurological, rheumatological, or severe musculoskeletal disease and a history of thoracic surgery were excluded. The patients in whom the exercise program was contraindicated (having an uncontrolled atrial/ventricular arrhythmia, systolic blood pressure ≥ 200 mm Hg or diastolic blood pressure ≥ 110 mm Hg at rest, severe aortic stenosis, recent embolism history, decompensated heart failure, recent cardiac ischemic event, an acute exacerbation of COPD) and patients changed their medication for COPD in the past month were also excluded.

### 2.2. Assessments

Demographics such as age, sex, body mass index, disease duration, and smoking status were recorded. The overall chest expansion was measured around the level of the nipple at the end of the deep inspiration and expiration, and the difference between the measurements was recorded [14]. The dyspnea severity of the patients was evaluated by the modified Medical Research Council (mMRC) dyspnea scale [15]. This scale measures both the level and functional impact of dyspnea and perceived limitations. It consists of five grades in which grade 1 is defined as no respiratory distress except with strenuous exercise, and grade 5 is defined as dyspnea too severe to leave the house or becoming out of breath while undressing.

The disease-specific quality of life of patients was assessed with the validated Turkish version of St. George’s Respiratory Questionnaire (SGRQ) [16]. This questionnaire has three components: symptoms, activity, and impact of the disease on activities of daily living. Items were scored from 0 to 100, where 0 indicates the best and 100 shows the worst state of health [17].

#### 2.2.1. Pulmonary function tests

Dynamic pulmonary function tests (PFT) were measured (Vyntus CPX Metabolic Cart, Vyaire Medical) at baseline and after the 6-week PR program by an experienced physiotherapist (SO) following the recommendations in guidelines [18]. The vital capacity (VC, L), forced vital capacity (FVC, L), forced expiratory volume in one second (FEV1, L), FEV1/FVC, peak expiratory flow (PEF, L/min), maximal expiratory flow 50 (MEF50, L/min), and maximal voluntary ventilation (MVV, L/min), and predicted values for each measurement were recorded.

#### 2.2.2. Cardiopulmonary exercise testing (CPET)

The cardiopulmonary exercise test was conducted on a treadmill using the modified Bruce protocol to determine the exercise capacity of each participant and the target exercise intensity during PR by the same physician (AG) and the same physiotherapist (SO). Twelve-lead electrocardiography (EKG Master, Tepa Medical, Ankara), blood pressure, and breath-by-breath analysis of respiratory gases (Vyntus CPX Metabolic Cart, Vyaire Medical) were monitored and recorded at the baseline, during, and after the exercise testing. The resting and maximal heart rates, exercise duration, systolic and diastolic blood pressures, maximal oxygen consumption during exercise (VO_2max_, ml/kg/min), oxygen consumption at anaerobic threshold (ml/kg/min), and metabolic equivalent (MET) values were recorded.

#### 2.2.3. 6-min walk test (6MWT)

A 6MWT was performed in a 30 m long inside hallway following standard guidelines [19].

#### 2.2.4. Ultrasonographic evaluation

Ultrasonographic evaluation (B-mode) of the diaphragm was performed using a linear array transducer (7- to 12-MHz) with an ultrasound scanner (LogiqP5, GE Medical Systems), by a physical medicine and rehabilitation specialist (SG) with more than 9 years of experience in musculoskeletal ultrasonography who was blinded to patients’ other assessments. All examinations were performed while the patient was in a supine position, and the transducer was placed longitudinally on the chest wall in the eighth or ninth right intercostal space between the anterior axillary and mid-axillary line [8,20]. The diaphragm was identified by its characteristic three-layered appearance composed of two hyperechoic lines of pleural and peritoneal fascia and hypoechoic muscle tissue in between. The diaphragm thickness (dt) measurements were done at the end of expiration (at Functional Residual Capacity (dt FRC)) and the end of the maximal inspiration (at Total Lung Capacity (dt TLC)) [6] at the point where the diaphragm became obscured by the lung (Figure). The average value of three consecutive measurements was recorded. This technique was previously reported to be reliable, with intraclass correlation coefﬁcients ranging from 0.89 to 0.98 for intra-rater and inter-rater reliability [21]. The thickening ratio (dtr) was calculated by dt TLC/dt FRC for determining contractility [6].

#### 2.2.5. Pulmonary rehabilitation

All subjects participated in an out-patient based PR program including controlled breathing techniques and respiratory strategies, muscle strengthening, flexibility and stretching, posture and aerobic exercises. The aerobic exercise program was individualized according to each patient’s CPET results and exercise capacity. A moderate-intensity exercise at 60%–70% of the patient’s maximal heart rate and/or 50%–60% of VO_2max_ was targeted. The aerobic exercise included walking on the treadmill for 3 sessions (40–50 min each) per week for 6 weeks. All exercises were performed under the supervision of the physiotherapist (SO). In the framework of PR, patients were educated about medical treatment (FÇ), breathing strategies, dietary regulation and consulted by a dietician when necessary, and psychological/behavioral support was arranged according to their needs.

All assessments were performed at baseline and after the completion of 6-weeks of PR.

#### 2.2.6 Statistical analysis

The R statistical program was used for statistical analysis. Numerical data were expressed as mean ± standard deviation, median (min-max), and categorical data were expressed as frequency (percentage). The Kolmogorov–Smirnov test was used to evaluate the normal distribution of data. A paired t-test/Wilcoxon test was used for interindividual changes due to parametric/nonparametric distribution. The independent sample t test was used to measure differences between two groups. The Pearson/Spearman correlation coefficient was calculated to evaluate the correlation between variables. The p value <0.05 was considered statistically significant.

## 3. Results

A total of 63 COPD patients were referred for the study. After the assessment for eligibility, 34 patients were included. The mean age and disease duration of the patients were 61.0 ± 8.2 years, 45 ± 70.2 months respectively. The mean FEV1% predicted was 57.9 ± 20.4, and most of the patients (47%) had moderate COPD according to the predicted FEV1 range. None of the patients were using systemic corticosteroids or in the need of oxygen therapy. The demographic and clinical characteristics of the subjects are presented in Table 1.

All patients showed significant improvements after PR regarding 6MWT, SGRQ (impact and total scores), chest expansion, and all CPET parameters. Among the PFT measures, only VC and MEF 50 improved significantly (Table 2). The ultrasonographic diaphragm assessments were found to be similar in two patient groups when they were classified as mild/moderate and severe/very severe according to the FEV1 range. While there was a significant increase in dt FRC values after PR in both groups, this increase did not differ between groups (Table 3). A positive correlation between the difference in dt FRC and the difference in 6MW distance after PR was found (rho = 0.552 p = 0.004). No further correlations were observed between diaphragm thickness changes and other parameters.

Considering that there is no similar study conducted before, power analysis couldn’t be performed, and the sample size couldn’t be determined at the beginning of the study. However, the correlation coefficient of pre and postrehabilitation dt FRC differences were found to be r = 0.77. Accordingly, the effect size was calculated to be 0.56 using a paired t-test, and the power of the study was 92% with 34 patients. Also, the correlation coefficient of pre and postrehabilitation dt TLC difference was found to be r = 0.75, and the effect size was found to be 0.47 using paired t-test, which makes the power of the study 88%.

## 4. Discussion

Results of this study demonstrate that 6 weeks of PR causes an increase in diaphragm thickness in patients with COPD regardless of the disease severity without any significant improvement in diaphragm contractility. To the best of our knowledge, this is the first study investigating the effects of PR on diaphragm thickness and contractility in patients with COPD.

In a previous study, the diaphragm thicknesses in COPD patients were found to be similar to those of healthy controls with the representative value of dt FRC ranging from 1.4 to 3.6 mm in patients and 1.6 mm to 3.4 mm in healthy adults [5]. Although it was reported that the ultrasonographic diaphragm thickness was significantly correlated with the trans-diaphragmatic pressure, De Bruin et al. evaluated the thickness of the diaphragm in boys with Duchenne muscular dystrophy and observed increased thickness despite the decreased contractility possibly due to pseudohypertrophy [22]. Therefore, it has been stated that the ultrasonographic thickening ratio should be evaluated since the diaphragm contraction is maximal at the full inspiration that is considered as an indicator of contractility. In this context, Boon et al. showed that diaphragm contractility doesn’t change significantly with age and sex, and the normal thickening ratio is within the range of 1.2–1.8, where values below 1.2 may indicate abnormality [6]. Considering these findings and the limitations involved in the current study, it may be stated that the prerehabilitation diaphragm thickness and thickening ratio were within normal limits regardless of the disease severity and did not indicate an obvious ultrasonographic diaphragmatic dysfunction in our study population. Similarly, Baria et al. [8], reported normal diaphragm contractility in patients with COPD (dtr:1.9 ± 0.5) compared to normal subjects (dtr: 1.8 ± 0.5), and Ogan et al. showed that there is no relationship between the disease severity and the thickness of the diaphragm [9].

Clanton and Levine suggested that diaphragm alterations in COPD are essentially due to the adaptive processes of a complex muscle responding to the changes to its mechanical environment rather than a form of dysfunction [23]. The authors also emphasized that if diaphragmatic alterations are apparent in some patients, the diaphragm might respond to treatment, which would be an important goal [23], and our results support this idea. Exercise training remains the only known intervention to reverse some of the underlying skeletal muscle abnormalities seen in COPD [24]. During muscular activity, the work and energy cost of breathing is increased [25]. This increased inspiratory work causes a metabolic load on the diaphragm, sometimes impairing diaphragmatic contraction in severe or acute cases, but still forceful breathing during exercise might be considered as a driving force to enhance contractibility of the diaphragm [24]. The moderate-high aerobic exercise training results in high ventilatory demand, and it was even stated that specific inspiratory muscle training might be unnecessary since the inspiratory muscles may already become trained by chronic intrinsic mechanical loading [24]. In this context, to our knowledge, there is only one study evaluating the effects of PR on the diaphragm thickness by using B-mode sonography [26]. The authors measured the distance, thickness, and length between the diaphragm and skin at FRC and TLC. However, the contractility of the diaphragm was not measured, which was stated as a limitation of the study. Their primary finding was an increase in diaphragm displacement observed after the PR. They also found that change in the length of the zone of apposition has the potential to predict the positive outcome of the exercise training program [26]. The different measurement methods of diaphragm properties make it difficult to compare the results of that study with ours. So, our study is the first one showing that PR leads to an increase in the diaphragm thickness in patients with COPD regardless of the disease severity. This finding is important to show that alterations in the diaphragm thickness during COPD can be modified with PR at every stage of the disease. Since the change in dt FRC was positively correlated only with the change in 6MW distance, it is yet to be further studied if the increase in diaphragm thickness has clinical significance or not. Although changes in 6MW distance with PR did not reach the level of accepted minimal clinically important difference for 6MWT, we consider this relation important, as the change in 6MW distance is an indicator of the functional performance improvement.

There are a couple of studies evaluating the effects of PR on the diaphragm [12,13]. However, in these studies, the craniocaudal mobility of the diaphragm was evaluated. Chun et al. used fluoroscopy and showed increased diaphragmatic mobility without significant improvement in PFT after PR [13]. Corbellini et al. also demonstrated an increase in diaphragmatic mobility by M-mode ultrasound only during deep inspiration after PR without any observed differences in normal breathing [12]. The authors emphasized that as air trapping increases in COPD, diaphragm excursion decreases, and they suggested that reducing dynamic lung hyperinflation with PR may lead to increased diaphragm mobility [12]. In this context, the decreased hyperinflation after PR might cause the diaphragm to seem ‘thicker’ since the diaphragm reverts into a better physiologic position. However, it was shown that decreasing lung volume from FRC to residual volume lengthens the diaphragm by about 5% [27]. Therefore, hyperinflation affects the length of the diaphragm rather than its thickness. Since the costal diaphragm fiber thickness was evaluated in this study, not the diaphragm excursion, reduced hyperinflation does not affect the results of the ultrasonographic measurement. Yet, the evaluation of the relationship between air trapping and diaphragm thickness may shed light on this under-researched field in future studies.

Contrary to what we expected, the diaphragmatic contractility was similar between groups, and no difference was observed after PR. The reason for no obvious change in contractility might be due to the absence of apparent diaphragmatic dysfunction in our study population. Besides, in the previous studies, dtr values were used to evaluate diaphragm function, but the significance of dtr to determine a treatment response is not well defined. Some authors state that dt FRC is more useful in serial studies in individual patients for comparison in the course of disease progression [28,29]. For these reasons, we think that the increase in dt FRC before and after rehabilitation beyond the standard error may be important for the improved diaphragm function even if dtr did not increase. Future studies including a healthy control group with longer PR courses might be better to evaluate dtr changes as a treatment response.

There is strong evidence that PR improves exercise capacity in terms of increased 6MW distance, VO_2peak_, quality of life, and breathlessness [10,11,30] in patients with COPD. The same results were observed in our study as well, besides, PR increased diaphragm thickness, which may contribute to the aforementioned effects of PR. These results demonstrated the effects of PR on the diaphragm, which is an important goal of PR.

There are some limitations to the study. First, this is not a randomized controlled study. As the present evidence shows that PR provides significant benefits to patients, it is thought that conducting a study with a non-intervention group would not be appropriate. To handle this ethical issue, to conduct a randomized controlled study with the patients when there is a long waiting list for PR and apply PR afterward to the nonintervention group might be an option. However, this isn’t the case in our facility since patients can be served immediately. Second, although this study aimed to evaluate possible changes in diaphragm thickness with PR in patients with COPD, a control group of healthy volunteers could provide information for comparison. Third, the study population was not homogenous regarding sex, consisting of mostly males. However, it was shown that diaphragm properties don’t change significantly with the sex [6]. Last, the wide range of the disease duration of the study population might result in a statistical error. Nevertheless, there was no correlation between disease duration and ultrasonographic diaphragmatic thickness parameters in this study.

In conclusion, our study showed that the 6-week PR resulted in a significant increase in diaphragm thickness without significant differences in diaphragm contractility in patients with COPD. Future studies, after overcoming the limitations of this study, might provide a better understanding of the effect of PR on the diaphragm and its resultant effects on the individual’s functionality.

## Figures and Tables

**Figure:**
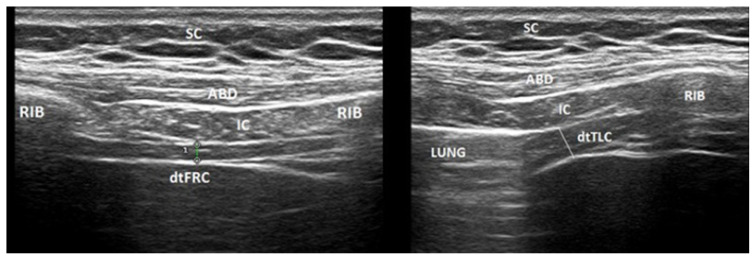
Ultrasonographic diaphragmatic thickness measurement at expiration (dt FRC) and maximal inspiration (dt TLC). ABD: Abdominal muscles, dt FRC: Diaphragmatic thickness at functional residual capacity, dt TLC: Diaphragmatic thickness at total lung capacity, IC: Intercostal muscles, SC: Subcutaneous tissue.

**Table 1:** Demographic characteristics of the patients.

Variable	n = 34
Age, y	61.0 ± 8.2
	60 (42–76)
Sex, male (%)	29 (85.3)
BMI kg/m^2^	24.2 ± 5.3
Disease duration, mo	45 ± 70.2
	60 (2–240)
Smoking (none/current/former)	4/0/30
Smoking, pack-year	40.4 ± 24.1
FEV1 range n, %	
Mild (≥ %80)	5 (14.7)
Moderate (50%-79%)	16 (47.1)
Severe (30%–49%)	8 (23.5)
Very severe (< 30)	5 (14.7)
mMRC grades n, %	
Grade 0	11 (32.4)
Grade 1	14 (41.2)
Grade 2	3 (8.8)
Grade 3	5 (14.7)
Grade 4	1 (2.9)

Results were given as n (%), mean ± SD or median (range).

BMI: Body mass index, FEV1: Forced Expiratory Volume in 1 s, mMRC: Modified Medical Research Council.

**Table 2:** Patient clinical assessments before and after pulmonary rehabilitation.

	Prerehabilitation n = 34	Postrehabilitation n = 34	p value
mMRC grades	1 (0–4)	0.5 (0–4)	0.000
6 MWT, m	447.5 ± 95.5	474.2 ± 110.1	0.05
Chest expansion, cm	3 (0.5–5)	3 (1–7)	0.01
SGRQ scores			
Symptom	53.3 ± 19.3	50.9 ± 20.3	0.36
Activity	53.0 ± 29.2	48.4 ± 29.4	0.07
Impact	33.9 ± 20.9	27.0 ± 22.7	0.03
Total	42.8 ± 20.8	37.2 ± 21.5	0.01
PFT parameters			
VC max, L	3.2 ± 0.9	3.3 ± 0.9	0.03
VC max pred, %	84.1 ± 22.0	87.0 ± 23.1	0.02
FVC, L	2.7 ± 0.9	2.6 ± 0.9	0.73
FVC pred, %	75.3 ± 23.3	74.8 ± 22.3	0.77
FEV1, L	1.6 ± 0.6	1.6 ± 0.7	0.31
FEV1 pred, %	57.9 ± 20.4	56.7 ± 21.7	0.42
FEV1/FVC %	61.9 ± 12.9	61.4 ± 14.0	0.67
PEF, L	4.7 ± 1.7	4.8±2.0	0.49
PEF pred, %	61.7 ± 20.8	63.3 ± 23.9	0.53
MEF50^a^, L	0.82 (0.2–3.3)	0.70 (0.2–2.7)	0.02
MEF50^a^ pred, %	24 (4–67)	22 (6–68)	0.03
MVV, L	62.0 ± 19.9	64.5 ± 22.8	0.12
MVV pred, %	68.6 ± 25.8	71.1 ± 29.0	0.17
CPET parameters			
VO_2peak_^a^ (ml/kg/min)	18.5 (8.3–32.4)	21.05 (12.8–35.9)	0.00
VO_2peak_ (predicted%)	75.5 ± 24.9	82.0 ± 28.1 (10.8)	0.00
VE (L/min)	52.5 ± 14.1	57.5 ± 18.6 (%9.5)	0.01
VCO2^a^ (L/min)	1281 (611–2468)	1353 (593–2944)	0.00
RER	0.9 ± 0.1	1.0 ± 0.1	0.01

Results were given as mean ± SD or median (range).

CPET: Cardiopulmonary Exercise Test, FEV1: Forced Expiratory Volume in 1 second, FVC: Forced Vital Capacity, MEF: Maximal Expiratory Flow, mMRC: Modified Medical Research Council, MVV: Maximum Voluntary Ventilation, PEF: Peak Expiratory Flow, PFT: Pulmonary Function Tests, RER: Respiratory Exchange Ratio, SGRQ: St George’s Respiratory Questionnaire, 6 MWT: 6-min Walk Test, VC: Vital Capacity, VE: Minute Ventilation, Paired-t test, a: Wilcoxon Test.

**Table 3:** Ultrasonographic diaphragm thickness measurement before and after pulmonary rehabilitation according to the FEV1 range.

	Mild-moderate			Severe-very severe			p value (within groups) ^a^
	Before n = 20	After n = 20	p value	Before n = 14	After n = 14	p value	
dt FRC (mm)	1.8 ± 0.2	1.9 ± 0.2	0.03	1.9 ± 0.5	2.2 ± 0.5	0.02	0.18
dt TLC (mm)	4.0 ± 1.0	4.3 ± 0.8	0.06	4.1 ± 0.9	4.4 ± 0.9	0.09	0.86
dtr	2.1 ± 0.5	2.1 ± 0.5	0.92	2.0 ± 0.5	2.0 ± 0.5	0.81	0.76

Results were given as mean ± SD, dt FRC: Diaphragm thickness at Functional Residual Capacity, dt TLC: Diaphragm thickness at Total Lung Capacity, dtr: Diaphragm thickening ratio Paired-t Test, a: Independent t-Test.
